# Prevalence of chronic kidney disease and metabolic related indicators in Mianzhu, Sichuan, China

**DOI:** 10.3389/fpubh.2023.1252110

**Published:** 2024-01-17

**Authors:** Feng Chen, Miao Wang, Yan Jiang

**Affiliations:** ^1^Department of Integrated Care Management Center, West China Hospital, Sichuan University, Chengdu, Sichuan, China; ^2^Public Affairs Department, West China Hospital, Sichuan University, Chengdu, Sichuan, China; ^3^Department of Nursing/Evidence-based Nursing Center, West China Hospital, Sichuan University, Chengdu, Sichuan, China

**Keywords:** chronic kidney disease, diabetes, hypertension, hyperlipidemia, body mass index

## Abstract

**Background:**

Chronic kidney disease (CKD) is a major public health problem worldwide. Periodic surveys are essential for monitoring the prevalence of CKD and its risk factors. We assessed the prevalence of CKD and its risk factors in Mianzhu City in 2020.

**Method:**

The Natural Population Cohort Study surveyed 7,770 individuals aged>20 years in Mianzhu City of Sichuan province in 2020. Our investigation encompassed the measurement of CKD prevalence, the evaluation of various renal function indicators, and comparisons based on age, gender, and hukou status. Additionally, some metabolic indices were also measured to identify the underlying causes of CKD.

**Results:**

(1) Overall, the prevalence of reduced renal function (eGFR<60 mL/min/1.73m^2^), albuminuria, and CKD were 1.3, 10.0, and 10.4%, respectively, (2) the overall prevalence of CKD was higher among men than among women (14.5% vs. 8.6%). Similarly, the prevalence of CKD was higher among men than women in most age groups, (3) among urban residents, the prevalence of CKD was higher among middle-aged individuals and lower among young individuals and older adults, and (4) considering eGFR, the albuminuria and CKD for group definition, Blood pressure, triglyceride, high-density lipoprotein, blood sugar, and BMI were all statistically different among between normal groups and abnormal groups s in the albuminuria and CKD.

**Conclusion:**

The incidence of CKD greatly varied between Mianzhu City and other regions in China and other countries. The differences in risk factors of CKD should be explored in the future. The gender difference in the prevalence of CKD in this study was markedly different from that in previous studies. More high-quality studies are needed to further explore this controversy. Based on the different prevalence of CKD and metabolism-related indices in rural and urban areas in this study, we speculated that the high incidence of CKD in Mianzhu City might be related to diet, lifestyle, and availability of healthcare services.

## 1 Introduction

Chronic kidney disease (CKD) is a progressive condition characterized by structural and functional changes in the kidney. Chronic kidney disease is typically defined as reduced kidney function, estimated glomerular filtration rate (eGFR) of less than 60 mL/min per 1.73m^2^, or imaging evidence or laboratory markers of kidney damage, such as albuminuria, hematuria that persist at least for 3 months (1). CKD is globally recognized as a public health problem. It is associated with an increased risk of end-stage kidney disease. Additionally, it is an independent risk factor for cardiovascular diseases and premature death (2, 3). The prevalence and associated burden of CKD are rising worldwide, with the fastest growth occurring in low-income and middle-income countries (4–6). According to an epidemiological survey in 2012, the overall prevalence of CKD was 10.8% in China. The number of Chinese patients with CKD is estimated to be nearly 119.5 million (5).

CKD is usually insidious, and most affected individuals remain asymptomatic until the disease progresses. Early detection and secondary prevention of CKD by modifying major risk factors and using inexpensive medications are cost-effective solutions for developing countries. Hyperglycemia, hypertension, hyperlipidemia, and obesity are major contributors to the global burden of disease and are the most common risk factors of CKD (7–11). Therefore, in addition to determining the kidney function of the general population, we explored the prevalence of these risk factors in this study. We identified individuals at high risk of CKD, which can help implement preventive strategies.

This study was conducted based on data from the Natural Population Cohort Study (NPCS). Previous studies have indicated that the prevalence of CKD substantially differs between different regions worldwide (12–14). This heterogeneity might be related to differences in lifestyles and economic conditions (15, 16). Mianzhu is close to Chengdu, the capital city of Sichuan province. Because of its poor economy and frequent natural disasters, it was included in many medical investigations (17–19). We collected the demographic data of residents in Mianzhu from the NPCS. We preliminarily measured the renal function of residents in Mianzhu, which can primarily help design subsequent cohort studies.

## 2 Methods

### 2.1 Participants

Data for this study were derived from NPCS. The NPCS is a population-based multicenter cohort study conducted by the Clinical Research Group at WCH/SCU. This study was approved by the Ethics Committee of the WCH/SCU [NO. (2020)145] and the Ministry of Science and Technology of China [No. (2020) CJ0150]. This study used the cluster sampling method (community or village) to recruit participants. Adult participants and their relatives who (1) aged ≥20 years and (2) had local household registration or non-local household registration but lived in the area for more than 6 months were invited to participate. The NPCS began in 2020 and will continue for at least 5 years.

### 2.2 Data collection

A questionnaire was used to collect demographic characteristics, such as gender, age, marital status, education, health insurance, commercial insurance, smoking, and alcohol drinking. Data were collected by face-to-face interviews with respondents and their family members. Height and weight were measured by nurses in the field. The subjects were asked to sit still for 20 min before blood pressure measurement. The left upper limb was taken for measurement with the help of trained evaluators. Blood pressure was measured between 8 and 10 a.m. with an empty stomach. Blood samples (4 mL in ethylenediaminetetraacetic acid (EDTA; plasma) and 1.5 mL in serum separator tube (SST; serum)) were collected for measuring complete blood count between 8 and 10 a.m. with an empty stomach. Random urine samples were collected between 8 and 10 a.m.

### 2.3 Laboratory measurement and specimen collection

(1) Chronic kidney disease (CKD) was defined as eGFR less than 60 mL/min per 1.73m^2^ or the presence of albuminuria (20). We used CKD-EPI to estimate eGFR. According to the Kidney Disease Outcomes Quality Initiative (K/DOQI) guidelines, we divided eGFR into five levels. eGFR class one was defined as eGFR ≥60 and was considered normal kidney function; eGFR class two was defined as eGFR between 45 and 59 and was considered a mild decline in kidney function; eGFR class three was defined as eGFR between 30 and 44, and was considered a moderate decline in kidney function; eGFR class four was defined as eGFR<30, and was considered a severe decline in kidney function (21).

Albuminuria was classified into -, +, ++, and +++ in urine analysis. Albuminuria ≥1+ was considered positive.

(2) Hypertension was defined as the presence of average systolic blood pressure ≥ 140 mmHg or diastolic blood pressure ≥ 90 mmHg, previous diagnosis of hypertension, or use of antihypertensive medication. Hypertension was divided into three stages: stage 1 hypertension (SBP 140–159 mmHg and/or DBP 90–99 mmHg); stage 2 hypertension (SBP 160–179 mmHg and/or DBP 100–109 mmHg); and stage 3 hypertension (SBP>180 mmHg and/or DBP>110 mmHg) (22).

(3) Diabetes was defined based on the following criteria: average fasting blood glucose (FBG) ≥ 7.0 mmol/L, currently taking anti-diabetic medications, or the previous diagnosis of diabetes (23).

(4) Dyslipidemia was defined as an abnormal lipid profile characterized by increased levels of total cholesterol (TC), low-density lipoprotein cholesterol (LDL-C), and triglyceride (TG) and decreased levels of high-density lipoprotein cholesterol (HDL-C). The specific criteria were as follows: TC higher than 6.2 mmol/L; LDL-C higher than 4.1 mmol/L; TG higher than 2.3 mmol/L; and HDL-C lower than 1.0 mmol/L (24, 25).

### 2.4 Statistics analysis

We excluded 869 participants with missing values. In addition, all participants (156) from a survey site with extremely poor-quality data on blood pressure were excluded, leaving 7,770 individuals for final analysis.

Demographic characteristics of patients and risk factors of CKD were determined. Quantitative variables are presented as mean and standard deviation (SD), and qualitative variables are presented as numbers and percentages. We used the χ^2^ test and t-test to compare groups. All analyzes were performed using SPSS version 25. value of p (two-tailed)<0.05 was considered statistically significant.

## 3 Results

### 3.1 Demographic characteristics

Of 7,770 participants, 35.8% were men and 64.2% were women. Most of the participants were > 45 years old and married. Their education level was mostly junior high school or below. The majority of urban and rural residents had health insurance, but most of them did not have commercial insurance. In addition, 15% of participants were current smokers and 20% drank alcohol. Except for commercial insurance, other variables were significantly and differently distributed among males and females (Table 1).

**Table 1:** Characteristics of participants.

	Total (%)	Male (%)	Female (%)	*p*
*Age groups (years)*				**<0.001**
22–34	130 (1.7)	51 (39.2)	79 (60.8)	
35–44	310 (4.0)	69 (22.3)	241 (77.7)	
45–54	1,657 (21.3)	387 (23.3)	1,271 (76.7)	
55–64	2096 (27.0)	664 (31.7)	1,432 (68.3)	
65–74	2,681 (34.5)	1,151 (42.9)	1,530 (57.1)	
75+	895 (11.5)	462 (51.6)	433 (48.4)	
*Marital status*				**<0.001**
Married (living together)	6,863 (88.3)	2,606 (38.0)	4,257 (62.0)	
Unmarried/divorced/separation/widowed	907 (11.7)	177 (19.5)	730 (80.5)	
*Education*				**<0.001**
Primary school or below	3,874 (49.9)	1,304 (33.7)	2,570 (66.3)	
Junior high school	2,757 (35.5)	1,010 (36.6)	1747 (63.4)	
Senior high school	841 (10.8)	328 (39.0)	513 (61.0)	
College or above	298 (3.9)	141 (47.3)	157 (52.7)	
*Medical insurance*				**<0.001**
Medical insurance for urban workers	2054 (26.4)	847 (41.2)	1,207 (58.8)	
Health insurance for urban and rural residents	5,438 (70.0)	1835 (33.7)	3,603 (66.3)	
No insurance	278 (3.6)	101 (36.3)	177 (63.7)	
*Commercial insurance*				0.275
Yes	240 (3.1)	78 (32.5)	162 (67.5)	
No	7,530 (96.9)	2,705 (35.9)	4,825 (64.1)	
*Smoking*				**<0.001**
Yes	1,358 (17.5)	1,168 (86.0)	190 (14.0)	
No	5,844 (75.2)	1,112 (19.0)	4,732 (81.0)	
Quit	568 (7.3)	503 (88.6)	65 (11.4)	
*Drinking*				**<0.001**
Yes	2,264 (29.1)	1,467 (64.8)	797 (35.2)	
No	5,093 (65.5)	1,034 (20.3)	4,059 (79.7)	
Quit	413 (5.3)	282 (68.3)	131 (31.7)	
*Total*	7,770 (100)	2,784 (35.8)	4,986 (64.2)	

Significant value are in bold, *p*<0.05.

*p*-value was based on Chi-square test.

### 3.2 Renal function tests

Table 2 shows that only 1.3% of participants had abnormal eGFR [<60 mL.min^−1^(1.73 m^2^)^−1^]. Most of them had an eGFR of between 45 and 59 mL.min^−1^(1.73 m^2^)^−1^. In addition, 10.1% of participants had positive urine protein tests. We found that 10.4% of participants had CKD. Decreased eGFR, positive urine protein result, and CKD were all more common among men. Positive urine protein and CKD were statistically different between men and women (*P*<0.001).

**Table 2:** Kidney function of participants.

	Total (%)	Male (%)	Female (%)	*p*
*eGFR mL.min^−1^ (1.73 m^2^)^−1^*				
≥60	7,668 (98.7)	2,736 (98.3)	4,932 (98.9)	0.139
45 ~ 59	77 (1.0)	36 (1.3)	41 (0.8)	
30 ~ 44	16 (0.2)	8 (0.3)	8 (0.2)	
<30	9 (0.1)	3 (0.1)	6 (0.2)	
*Urine protein*				**<0.001**
−	6,993 (90.0)	2,402 (86.3)	4,591 (92.1)	
+	664 (8.6)	326 (11.7)	338 (6.8)	
++ or more	113 (1.5)	55 (2.0)	58 (1.2)	
*CKD*				**<0.001**
Normal	6,935 (89.6)	2,379 (85.5)	4,556 (91.4)	
Abnormal	835 (10.4)	404 (14.5)	431 (8.6)	
*Total*	7,770 (100)	2,783 (35.8)	4,987 (64.2)	

Significant value are in bold, *p*<0.05.

*p*-value was based on Chi-square test.

### 3.3 The prevalence of CKD by gender and hukou

**Figure 1:**
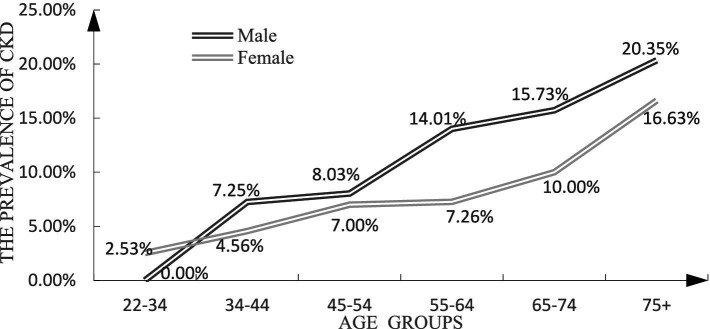
The prevalence of CKD by gender.

Figure 1 shows that the prevalence of CKD was higher among males than among females in most age groups. The gap between males and females gradually decreased in older ages.

**Figure 2:**
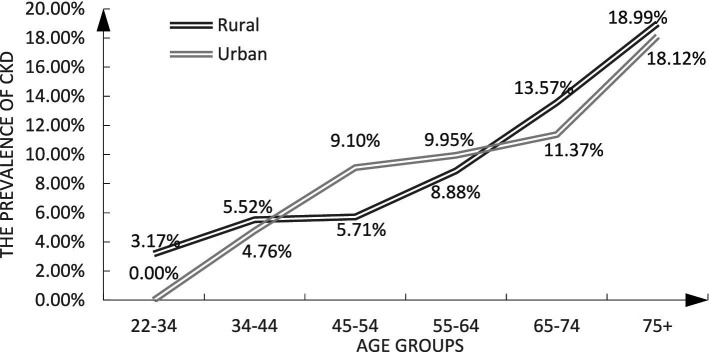
The prevalence of CKD by hukou.

Figure 2 illustrates that among urban residents, the prevalence of CKD was elevated among middle-aged participants and declined among both younger and older individuals.

### 3.4 The relationship between indicators of kidney injury and risk factors of CKD

Table 3 indicates that 34% of participants suffered from hypertension, 8.7% of participants suffered from prediabetes, 1.8% of participants suffered from diabetes, and 10.8% of participants had a BMI of more than 28.

**Table 3:** The relationship between indicators of kidney injury and risk factors of CKD.

		Total (%)	eGFR			Proteinuria			CKD		
			≥60 mL.min^−1^ (1.73 m^2^)^−1^	<60 mL.min^−1^ (1.73 m^2^)^−1^	*p*	Normal (%)	Abnormal (%)	*p*	Normal (%)	Abnormal (%)	*p*
Blood pressure	Normal	5,127 (66.0)	5,062 (98.7)	65 (1.3)	0.932	4,717 (92)	410 (8.0)	<0.001	4,680 (91.3)	447 (8.7)	<0.001
	Stage 1	1891 (24.3)	1865 (98.6)	26 (1.4)		1,655 (87.5)	236 (12.5)		1,640 (86.7)	251 (13.3)	
	Stage 2	646 (8.3)	637 (98.6)	9 (1.4)		539 (83.4)	107 (16.6)		534 (82.7)	112 (17.3)	
	Stage 3	106 (1.4)	104 (98.1)	2 (1.9)		82 (77.4)	24 (22.6)		81 (76.4)	25 (23.6)	
TC		7,770 (100.0)	5.098 ± 0.966	4.975 ± 1.130	0.200	5.096 ± 0.965	5.111 ± 0.996	0.673	5.096 ± 0.964	5.106 ± 0.997	0.762
TG		7,770 (100.0)	1.713 ± 1.289	1.911 ± 1.086	0.125	1.684 ± 1.259	2.005 ± 1.489	**<0.001**	1.683 ± 1.261	1.990 ± 1.458	**<0.001**
LDL-C		7,770 (100.0)	2.907 ± 0.745	2.872 ± 0.813	0.637	2.902 ± 0.743	2.941 ± 0.779	0.167	2.902 ± 0.742	2.939 ± 0.778	0.178
HDL-C		7,770 (100.0)	1.571 ± 0.352	1.465 ± 0.397	**0.001**	1.578 ± 0.349	1.496 ± 0.367	**<0.001**	1.578 ± 0.349	1.496 ± 0.362	**<0.001**
Hyperglycemia	Normal	6,958 (89.5)	6,883 (89.8)	75 (73.5)	**<0.001**	6,361 (91.0)	597 (76.8)	**<0.001**	6,315 (91.1)	643 (77.0)	**<0.001**
	prediabetes	676 (8.7)	656 (8.6)	20 (19.6)		519 (7.4)	157 (20.2)		512 (7.4)	164 (19.6)	
	Diabetes	136 (1.8)	129 (1.7)	7 (6.9)		113 (1.6)	23 (3.0)		108 (1.6)	28 (3.4)	
BMI	<18.5	201 (2.6)	201 (2.6)	0 (0.0)	**<0.001**	180 (2.6)	21 (2.7)	**<0.001**	180 (2.6)	21 (2.5)	**<0.001**
	18.5 ~ 23.9	3,766 (48.5)	3,730 (48.6)	36 (35.3)		3,463 (49.5)	303 (39.0)		3,443 (49.6)	323 (38.7)	
	24 ~ 27.9	2,966 (38.2)	2,923 (38.1)	43 (42.2)		2,633 (37.7)	333 (42.9)		2,607 (37.6)	359 (43.0)	
	>28	837 (10.8)	814 (10.6)	23 (22.5)		717 (10.3)	120 (15.4)		705 (10.2)	132 (15.8)	
Total		7,770 (100.0)	7,668 (98.7)	102 (1.3)		6,993 (90.0)	777 (10.0)		6,935 (89.3)	835 (10.7)	

Significant value are in bold, *p*<0.05.

*p*-value was based on chi-square test for qualitative variables.

*p*-value was based on t-test test for quantitative variables.

eGFR, Estimated glomerular filtration rate; CKD, Defined as eGFR<60 mL/min/1.73 m^2^ or evidence of proteinuria; TC, Total cholesterol; TG, Triglyceride; LDL-C, Low-density lipoprotein cholesterol; HDL-C, High-density lipoprotein cholesterol.

Hypertension was positively correlated with proteinuria and CKD (*p*<0.001). In addition, hypertension was positively correlated with the prevalence of patients with eGFR<60 mL.min^−1^(1.73 m^2^)^−1^, but the difference was not statistically significant (*p*>0.05).

Higher levels of TC, TG, and LDL-C and lower levels of HDL-C were observed among participants with proteinuria and CKD. TC, LDL-C, and HDL-C levels were lower in participants with eGFR<60. TG levels were higher in participants with normal eGFR<60. However, statistical significance was only observed for TG and HDL-C (*p*<0.001).

Prediabetes and diabetes were more common among patients with eGFR<60 mL.min^−1^(1.73 m^2^)^−1^, proteinuria, and CKD (*p*<0.001). Abnormal eGFR, proteinuria, and CKD were all more frequent in patients with BMI>24 than in the normal population (*p*<0.001).

Prediabetes, diabetes, and BMI were all more common among those with decreased eGFR, proteinuria, and CKD, and there was a significant difference between groups (*p*<0.001).

## 4 Discussion

In this study, there were more women than men, and most participants were > 50 years old. This may be due to the fact that men constitute the main proportion of the labor force, and older adults people and women are neglected in the non-developed cities of China. The educational level of participants was not high, and 85.3% of them had only completed junior high school or less. The coverage rate of health insurance in Mianzhu City was similar to that reported by a national survey (96.42% vs. 96.45%) (26). We found that only 3% of participants had commercial insurance. According to the 2020 China Household Wealth Index Survey Report, 10.8% of Chinese households are covered by commercial insurance (27). The low rate of commercial insurance indicates that the economy of Mianzhu is not prosperous.

We collected data on CKD stage, albuminuria, and eGFR. The prevalence of reduced renal function (eGFR<60 mL/min/1.73 m^2^) was 1.3%, the prevalence of albuminuria was 10.0%, and the overall prevalence of CKD was 10.4% in this study. We found that only the prevalence of reduced renal function in Mianzhu was lower than that in other regions. For instance, the prevalence of eGFR<60 mL/min/1.73m^2^, albuminuria, and CKD were 1.83, 8.65, and 9.88% in Zhejiang province, respectively (28). Using the same definition of CKD, a cross-section national survey in China showed that the prevalence of CKD was 10.8%, and the highest prevalence of CKD was found in Southwest China (18.3%) (20). The global estimated prevalence of CKD is 13.4% (29). CKD has various risk factors, including developmental, physical, social, cultural, structural, environmental, and genetic factors (30). Therefore, the difference in the prevalence of CKD between Mianzhu city and other regions may be due to the different prevalence of these risk factors.

Consistent with studies from Japan and Singapore (31, 32), the prevalence of CKD was higher among men than among women in our study. However, in most other geographical regions, the prevalence of CKD was higher among women (33–36). Some studies showed that despite including sex in the estimation, the CKD-EPI formula may overestimate eGFR in women (37). We used this formula, but the prevalence of CKD among men was still higher than that among women in this study. Chronic kidney disease resulting from hypertension or diabetes is more prevalent in men while autoimmune kidney disease or infectious urinary tract disease prevail in female (38). And in this study, we found that some metabolic risk factors, such as high blood pressure, dyslipidemia, diabetes, and high BMI were more common among men. Similarly, some national studies reported that the prevalence of hypertension (39), hyperglycemia (40), dyslipidemia (41), and obesity (42) were all higher among men than among women. We hypothesized that CKD is closely associated with metabolic factors in Mianzhu. In addition, as in Ciarambino’s study, the physiological differences in tubular absorption and secretory function between men and women may also account for the higher prevalence of CKD in men (38).

Among middle-aged residents, the prevalence of CKD was lower in rural areas than in urban areas. The main career of Minazhu citizens is agriculture, lacking a prosperous economy. Therefore, many rural residents migrate in their early adulthood to obtain better jobs. Existing studies have shown that the incidence of CKD is related to the Western lifestyle (15). Migrant workers do not have the sufficient economic power to maintain a high-oil, high-fat, and high-sugar diet and are, therefore, less likely to become obese and metabolically ill. Old immigrants return to the countryside, where public health services and medical resources are not abundant, which may increase the risk of CKD.

## 5 Limitation

We lost some participants who were migrant workers. Minority groups avoided participation due to their living habits and religious beliefs. In addition, there are many combinations of indicators for CKD. In this study, we referred to the previous large-scale cross-sectional survey on the prevalence of kidney disease in China, which only used eGFR and proteinuria to evaluate CKD. Since our data comes from the natural population, we only collected on eGFR and proteinuria for a single time point. These limitations may affect the measured rates of CKD. In future cohort studies, we may need to improve the relevant indicators and plans to obtain more reliable data on the prevalence of CKD. Finally, because of the cross-sectional design, this study cannot demonstrate a causal relationship between metabolic risk factors and CKD.

## Data availability statement

The datasets used and analyzed during the current study are available from the corresponding author on reasonable request.

## Ethics statement

The studies involving humans were approved by Ethics Committee of West China Hospital, Sichuan University. The studies were conducted in accordance with the local legislation and institutional requirements. The participants provided their written informed consent to participate in this study.

## Author contributions

YJ and MW: conceptualization, methodology, and writing, review & editing. FC: formal analysis, data visualization, and writing the original draft. All authors contributed to the article and approved the submitted version.
